# Characterization of DXZ4 conservation in primates implies important functional roles for CTCF binding, array expression and tandem repeat organization on the X chromosome

**DOI:** 10.1186/gb-2011-12-4-r37

**Published:** 2011-04-13

**Authors:** Christine R McLaughlin, Brian P Chadwick

**Affiliations:** 1 Department of Biological Science, Florida State University, 319 Stadium Drive, 3076 King Building, Tallahassee, FL 32306-4295, USA

## Abstract

**Background:**

Comparative sequence analysis is a powerful means with which to identify functionally relevant non-coding DNA elements through conserved nucleotide sequence. The macrosatellite DXZ4 is a polymorphic, uninterrupted, tandem array of 3-kb repeat units located exclusively on the human X chromosome. While not obviously protein coding, its chromatin organization suggests differing roles for the array on the active and inactive X chromosomes.

**Results:**

In order to identify important elements within DXZ4, we explored preservation of DNA sequence and chromatin conformation of the macrosatellite in primates. We found that DXZ4 DNA sequence conservation beyond New World monkeys is limited to the promoter and CTCF binding site, although DXZ4 remains a GC-rich tandem array. Investigation of chromatin organization in macaques revealed that DXZ4 in males and on the active X chromosome is packaged into heterochromatin, whereas on the inactive X, DXZ4 was euchromatic and bound by CTCF.

**Conclusions:**

Collectively, these data suggest an important conserved role for DXZ4 on the X chromosome involving expression, CTCF binding and tandem organization.

## Background

Macrosatellites are a type of variable number tandem repeat (VNTR) that primarily differ from other VNTRs by the size of the individual repeat unit (from 2 to >12 kb) and restriction of the array to one or two locations in the genome [1]. To date, at least eight different macrosatellite arrays have been described in the human genome [1-6], although several others remain largely unexplored [1].

Among the human macrosatellites, the best characterized is D4Z4, located at the subtelomeric regions of chromosomes 4q35 [6] and 10q26 [7,8]. D4Z4 consists of a tandem array of 3.3-kb repeat units that can cover hundreds of kilobases at these chromosomal locations [6]. D4Z4 has been a major focus for research since a link was made between the array and facioscapulohumeral muscular dystrophy (FSHD) [6,9], an autosomal dominant disorder characterized by progressive atrophy of muscles in the face, shoulders and upper arms [10]. In almost all cases, FSHD onset is associated with contraction of the array at 4q35 to ten or fewer repeat units [6,9]. Contraction alone is not sufficient for disease onset, as pathogenesis is linked to a reduction in the number of D4Z4 repeat units on a defined haplotype termed 4qA161 [11]. Like other macrosatellites [1,2,5,12,13], D4Z4 is expressed [14]. Each D4Z4 monomer in the array contains an ORF [15] that encodes a double homeobox protein termed DUX4 [16]. Recent data indicate that transcripts originating from the most distal monomer are stabilized by a poly-adenylation signal located distal to the array [14,17], a feature that is only found on chromosomes with the 4qA161 haplotype [18]. These advances have focused attention on inappropriate expression of DUX4 as the likely molecular basis of the disease.

Not all macrosatellites are obviously protein coding [5], which then begs the question of what role they fulfil in genome biology. One such example is the X-linked macrosatellite DXZ4. DXZ4 is a tandem array of 3-kb repeat units located at Xq23 [3]. While several short ORFs are present within a monomer, none have any homology to known proteins and they therefore may simply be a consequence of the high GC DNA sequence content reducing the incidence of stop codons. Identification of the ORF in D4Z4 was facilitated by the presence of the homeobox motif [15,16], and further supported by conservation of the ORF through to rodents [19]. However, despite no clear ORF within a DXZ4 monomer, the array does adopt intriguing chromatin states in the context of X chromosome inactivation, which is the mammalian form of dosage compensation, a process that balances X-linked gene expression between the sexes by shutting down most gene expression from one of the two X chromosomes in females [20]. Gene silencing at the chosen inactive X chromosome (Xi) is achieved by repackaging the chromosome into facultative heterochromatin, including CpG island DNA hypermethylation [21,22], acquisition of covalent histone modifications associated with heterochromatin [23-26], and underrepresentation of euchromatic marks, including histone acetylation [27] and histone H3 dimethylated at lysine 4 (H3K4me2) [23]. Whereas most chromatin on the Xi adopts this new configuration, DXZ4 does not. CpG dinucleotides at DXZ4 are hypomethylated [3,12] and DXZ4 nucleosomes are characterized by H3K4me2 [12,28] as well as several other euchromatic markers [29] and the array is bound by the multi-functional zinc finger protein CCCTC binding factor (CTCF) [12,29]. DXZ4 at the Xi is readily detected as a signal of H3K4me2 within the hypo-H3K4me2 territory of the Xi at interphase [28,29], and as a distinct signal midway down the long arm of the chromosome at metaphase [28]. Facultative heterochromatin of the Xi is not a homogenous mass of like-chromatin, but is instead composed of at least two different types that occupy reproducible alternating bands along the Xi [30,31]. DXZ4 resides at the interface of two such bands [28], and therefore may have some role in organization of the Xi involving CTCF. In sharp contrast, DXZ4 CpG dinucleotides on the active X chromosome (Xa) and in males are hypermethylated [3,12] and nucleosomes are packaged into constitutive heterochromatin characterized by histone H3 lysine-9 trimethylation (H3K9me3) [12] and binding of heterochromatin protein 1 gamma (HP1γ) [32]. Therefore, human DXZ4 adopts alternative chromatin states on the Xa and Xi that differ from the surrounding chromosome.

Intriguingly, analysis of D4Z4 chromatin organization in FSHD patients revealed that the contracted allele adopted a more euchromatic organization [32,33] involving CTCF binding [34], hence resembling DXZ4 on the Xi [35]. Therefore, these findings highlight how investigating the biology of macrosatellites in general can provide insight into the function of other macrosatellites such as D4Z4 in the context of FSHD.

In order to further understand DXZ4, we sought to identify conserved DNA sequence and chromatin organization for the array by investigating the macrosatellite in other primates, and report our findings here.

## Results

### DXZ4 is a conserved X-linked macrosatellite repeat in Old World and New World monkeys

**Figure 1:**
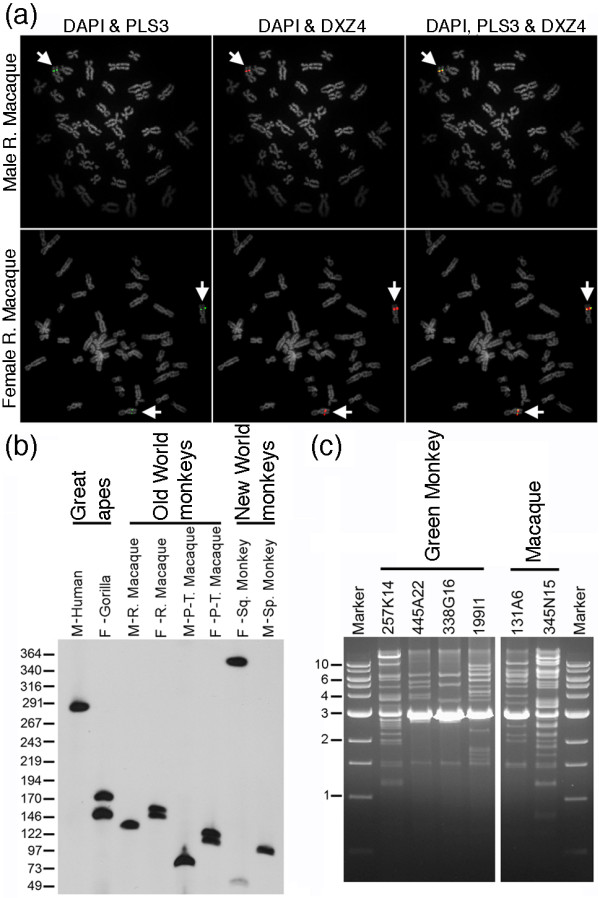
**Mapping and tandem arrangement of DXZ4 in primates**. **(a) **Direct-labeled fluorescence *in situ *hybridization (FISH) of human DXZ4 BAC clone (2272M5; red) and human *PLS3 *BAC clone (268A15; green) to male and female rhesus macaque metaphase chromosomes. White arrows point to the hybridizing X chromosome. Metaphase chromosomes were counterstained with DAPI, and converted to gray-scale to assist in visualizing the FISH signals. **(b) **Southern blot of *Xba*I digested primate genomic DNA separated by pulsed field gel electrophoresis, hybridized with a human digoxigenin-labeled DXZ4 probe. Primates and group are listed along the top and gender indicated by M (male) or F (female), including rhesus macaque (R. Macaque), pig-tailed macaque (P-T. Macaque), common squirrel monkey (Sq. Monkey) and black-handed spider monkey (Sp. Monkey). Size in kilobases is given to the left. **(c) **Ethidium bromide stained 0.9% agarose gel showing green monkey and macaque BAC DNA digested with the restriction endonuclease *Hin*dIII and separated by gel electrophoresis. The sizes of the molecular weight marker are given to the left in kilobases.

Previously, DXZ4 has been assigned to the X chromosome by fluorescence *in situ *hybridization (FISH) in gorilla, chimpanzee and orangutan [36], but nothing further is known about conservation of DXZ4 outside of the great apes. To determine if DXZ4 is sufficiently conserved in Old World monkeys, human probes for DXZ4 and the gene 70 kb proximal to the human array (*PLS3*) were hybridized to male and female rhesus macaque (*Macaca mulatta*) metaphase chromosomes. An intense signal for DXZ4 was detected on a single chromosome in the male sample and on two chromosomes in the female samples (Figure 1a). In both cases, the DXZ4 signal partially overlapped with the *PLS3 *signal. Collectively, these data indicate that DXZ4 in macaque is X-linked and its chromosomal location is conserved adjacent to *PLS3*.

Next we sought to determine if DXZ4 is a polymorphic tandem array in Old World monkeys and if DXZ4 sequence conservation was sufficiently high enough to detect DXZ4 in New World monkeys using a human DXZ4 probe. Agarose embedded genomic DNA from a human sample as a hybridization control, one female gorilla, two male macaques, two female macaques and a male and female New World monkey were cut with *Xba*I and fragments separated by pulsed field gel electrophoresis. Given that there are no recognition sites for *Xba*I in human DXZ4, genomic DNA will be cut at the first available sites proximal and distal to DXZ4, essentially excising the array intact. Because the copy number of DXZ4 monomers in humans varies between individuals, hybridizing DNA fragments are polymorphic [3]. A Southern blot of the gel was hybridized with the human probe before washing to high stringency (0.2 × SSC, 0.1% SDS at 60°C). A single hybridizing band was detected in all male samples, and two bands in the females, including the New World monkeys (Figure 1b). Three conclusions can be drawn from this result. First, DXZ4 sequence is conserved in primates at least as far as New World monkeys. Second, given the single male band and two female bands, this further supports X-linkage for DXZ4 in primates. Finally, the range of hybridizing fragments (approximately 50 to 350 kb) indicates that DXZ4 is a VNTR in the great apes, Old World monkeys and New World monkeys.

Comparison of the human DXZ4 DNA sequence [GenBank: HQ659140] against the rhesus macaque genome sequence (rheMac2), did not identify a VNTR, but instead identified various broken matches over short intervals and the presence of a large gap in the genome sequence, indicating that like many tandem repeat DNAs in the various early releases of the human genome, the sequence of DXZ4 is poorly assembled in the current build of the macaque genome. In order to further characterize DXZ4 in Old World monkeys, we compared the human DXZ4 sequence to entries in the public databases using BLAST. Several green monkey (*Cercopithecus aethiops*) and rhesus macaque BAC clones were identified with matches to DXZ4 that were then ordered and obtained. Human DXZ4 is cut once per monomer with *Hin*dIII [3]. Digestion of the Old World monkey BACs with *Hin*dIII revealed an over representation of a 3-kb fragment in the BAC clones (Figure 1c), supporting the presence of multiple DXZ4 sequences in the clones. The 3-kb *Hin*dIII band was excised from the gel and cloned before sequencing.

Comparison of either the complete 2,950 bp green monkey sequence [GenBank: HQ906499] or the complete 2,922 bp rhesus macaque sequence [GenBank: HQ906498] against human DXZ4 (2,994 bp) using ClustalW2 [37] revealed 87% nucleotide identity in both cases (Table 1). This is lower than the observed sequence divergence of 94% between the human and macaque X chromosomes [38]. Directly comparing the two Old World monkey DXZ4 sequences revealed 95% sequence identity.

**Table 1:** Comparison between human DXZ4 and monomer sequences identified in great ape, lesser ape, Old World monkey and New World monkey

Primate	Monomer size (bp)	Percent identity to human	GC content	CpG
Human	2,994	-	62%	186
Chimpanzee	2,996	97%	62%	176
Gorilla	2,954	95%	62%	165
Gibbon	2,896	90%	63%	166
Macaque	2,922	87%	62%	161
Green monkey	2,950	87%	61%	154
Marmoset	3,039	77%	65%	192

**Figure 2:**
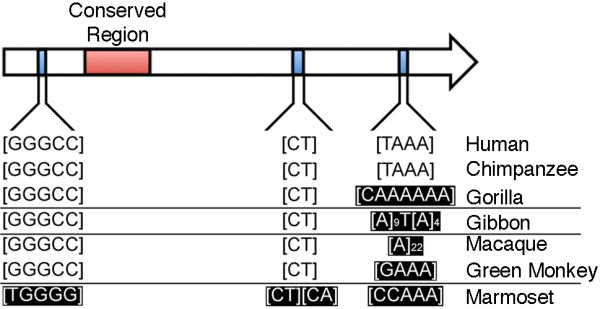
**Conservation of simple repeat sequences at DXZ4**. DXZ4 is represented by a right facing 3-kb monomer. Annotated on this monomer is the region of DXZ4 that is conserved in all primates examined (red box) as well as the location of the three internal simple repeat sequences (blue boxes), which account for all repetitive DNA in DXZ4. The sequence of the simple repeats in human is given immediately below the schematic map and beneath this the nucleotide composition in various other primates is given. Sequences that diverge from the human sequence are highlighted as white writing on a black background. The horizontal lines divide great apes from the lesser apes, Old World monkeys and New World monkeys.

Using the public databases, additional complete monomer sequences were extracted for chimpanzee, gorilla, white-cheeked gibbon (*Hylobates leucogenys*) and a New World monkey (*Callithrix jacchus *or the common marmoset) (Table 1). These sequences were then compared to human DXZ4. As expected, the percent nucleotide identity to human DXZ4 was highest within the great apes, lower in the lesser apes, lower still in Old World monkeys and lowest in New World monkeys. In all cases, the monomers were similar in size, GC content and the number of CpG dinucleotides. The DNA sequence of human DXZ4 is unique to its location on the X chromosome, with the exception of three internal polymorphic microsatellite sequences [3]; [GGGCC]n, [CT]n and [TAAA]n (Figure 2). In humans, copy number of these sequences accounts for most variation in DXZ4 between adjacent monomers (Tremblay DC *et al*., in preparation). In chimpanzee, all three of the microsatellite sequences are conserved. However, in gorilla the [TAAA] repeat is replaced by a [CAAAAAA] repeat, and by an A-rich sequence in gibbon (Figure 2). Both Old World monkey DXZ4 monomers contained 3 copies of [GGGCC], with 19 [CT] in rhesus macaque and 25 [CT] in the green monkey that were interrupted by two [GT]. Neither Old World monkey contained the [TAAA] repeat. Instead, the sequence was replaced with a 22-bp poly-A stretch in rhesus macaque, and 4 copies of [GAAA] in the green monkey (Figure 2). In the New World monkey, the [GGGCC] repeat is replaced by a [TGGGG] repeat. The [CT] repeat is still present, but immediately adjacent to this microsatellite is a new dinucleotide [CA] repeat. The [TAAA] repeat is also replaced by a [CCAAA] repeat. Collectively, these data indicate that the [TAAA] repeat is the least conserved internal repeat in primate DXZ4, but that all primates retain simple repeat DNA of similar base composition at these regions (Figure 2).

### Tandem repeat organization is retained in lemurs, but sequence conservation is restricted to the promoter and CTCF binding region of human DXZ4 in distantly related primates

**Figure 3:**
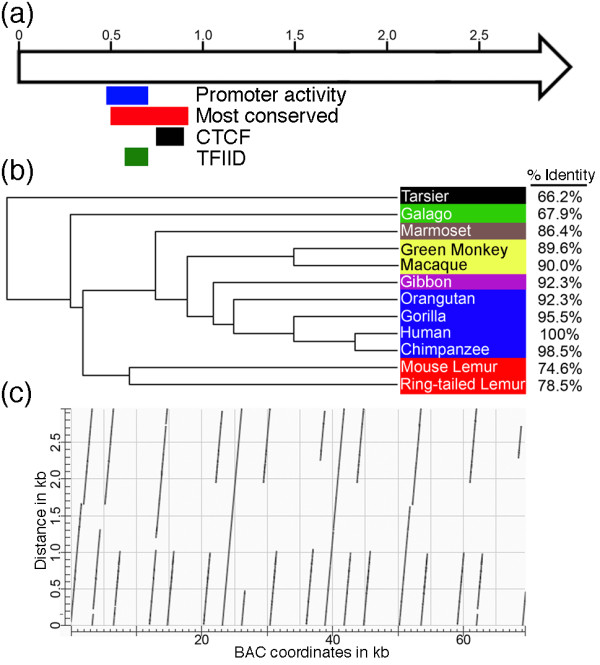
**DNA sequence conservation of DXZ4**. **(a) **Schematic map of a single human 3-kb DXZ4 monomer represented by an open arrow. Distance in kilobases is given along the top. Underneath, the colored bars indicate the regions of DXZ4 corresponding to the defined promoter, CTCF and TFIID binding sites [12] and the 402-bp interval of DXZ4 that is most conserved in primates. **(b) **Phylogenetic tree showing inferred evolutionary relationship of DXZ4 in primates. Sequence alignments were made against a 402 nucleotide region of human DXZ4 that is most conserved in all primates (nucleotides 517 to 918 of accession number [GenBank:HQ659140]). Percentage nucleotide identity is indicated to the far right. Primates are color-coded as follows: blue, great apes; pink, lesser apes; yellow, Old World monkeys; brown, New World monkeys; red, lemurs; green, galago; black, tarsier. The tree image was generated using MUSCLE version 3.8 [45]. **(c) **Predicted higher-order organization of the ring-tailed lemur DXZ4 sequence as revealed by dot-plot analysis. A single 3-kb DNA sequence is on the y-axis, whereas approximately 70 kb of BAC clone LB2-162N9 is given on the x-axis. The dot-plot was generated using the default settings for NCBI Blast2, and the output image labeled in Adobe Photoshop CS2.

The DNA sequence of human DXZ4 was used to identify homologous sequences from other primate entries in the public databases. Several matches were made with sequences from Sumatran orangutan (*Pongo pygmaeus abelii*), gray mouse lemur (*Microcebus murinus*), ring-tailed lemur (*Lemur catta*), small-eared galago (*Otolemur garnettii*) and the Philippine tarsier (*Tarsius syrichta*). However, for the more distantly related primates (lemurs, galagos and tarsiers), sequence matches were limited to a 402-bp interval of DXZ4 corresponding to the site where the epigenetic organizer protein CTCF is bound and bi-directional promoter activity has been assigned [12] (Figure 3a). Outside of this region, limited sequence homology can be detected, with the exception of an additional 326 bp that shares 69% broken homology with ring-tailed lemurs (data not shown). Using the most conserved 402-bp sequence from all primates, a phylogenetic tree was assembled (Figure 3b). The resulting cladogram is similar to one generated through investigation of mobile element distribution in primates [39]. Interestingly, sequence identity for this interval was higher than across the whole monomer through New World monkeys (compare Table 1 to Figure 3b), supporting this region as the most conserved part of DXZ4. It is important to note that the available sequence data for DXZ4 in many of the primates shown is limited. As more sequences are released into the public domain, it is conceivable that the phylogenetic relationship of at least the distantly related primates may change. Nevertheless, it is clear that sequence conservation across a single DXZ4 monomer breaks down outside of the New World monkey branch of the primate tree.

The ring-tailed lemur sequence was derived from a BAC clone (LB2-162N9) [GenBank: AC133072] within which a 170-kb continuous sequence is assembled. The first 70 kb of this sequence aligns with the 402-bp human DXZ4 sequence 15 times (data not shown), indicating a locally repetitive nature for this interval. Of the remaining 100 kb, multiple extensive single copy matches are made with unique DNA sequences found distal to human DXZ4, indicating that the BAC clone likely contains part of the orthologous ring-tailed lemur DXZ4 array. Comparison of the 170-kb BAC sequence against itself confirmed the presence of a more extensive tandem repeat in the first 70 kb than just the 402-bp sequence. Alignment of a 3-kb sequence from within this interval against the 70-kb sequence clearly demonstrates that the DNA has characteristics of a tandem repeat (Figure 3c). However, unlike human DXZ4 (Tremblay DC *et al.*, in preparation), the sequence is not a perfect tandem array of uninterrupted monomers of a uniform size, but consistent with other primates (Table 1), the 3-kb sequence is characterized by high GC content (62%).

### Conserved hypo-methylation of CpG residues on the macaque Xi

**Figure 4:**
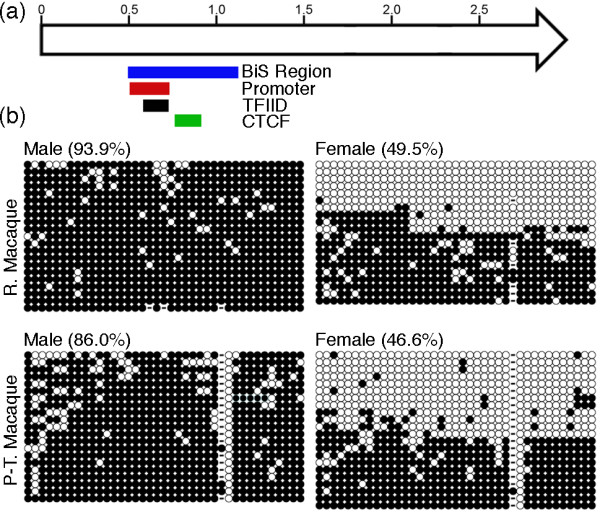
**DXZ4 CpG methylation patterns in male and female macaques**. **(a) **Schematic map of a single human 3-kb DXZ4 monomer represented by an open arrow. Distance in kilobases is given along the top. Underneath, the colored bars indicate the regions of DXZ4 corresponding to the defined promoter, CTCF and TFIID binding sites [12] and the 621-bp interval of macaque DXZ4 that was assessed for CpG methylation by bisulfite sequencing. **(b) **CpG methylation profile of the 621-bp region of macaque DXZ4 in male and female rhesus macaque (R. Macaque) and pig-tailed macaque (P-T. Macaque) samples. All 39 cytosine residues corresponding to CpG dinucleotides within the interval are represented by circles. White circles indicate no methylation whereas black circles indicate a methylated C. Each row of circles represents sequence data from an independent clone. Dashes within the profile represent a sequence that diverged from the consensus and did not have a C residue at that site. The given percentage of methylation is based on the number of methylated residues within the full profile.

In humans, DXZ4 on the male X and Xa in females is characterized by CpG methylation, whereas CpGs on the Xi are hypomethylated [3,12]. To determine if this was conserved at the macaque Xi, we prepared bisulfite modified genomic DNA from a male and female rhesus macaque and pig-tailed macaque primary fibroblast culture, and then proceeded to PCR amplify a 621-bp fragment spanning the region corresponding to the human promoter and CTCF binding site (Figure 4a). PCR products were TA cloned and at least 20 independent clones were sequenced. Consistent with humans, male DXZ4 is extensively methylated (Figure 4b, left panels). In contrast, female DXZ4 is hypomethylated in a little over half of all clones (Figure 4b, right panels). The logical interpretation of these data is that, like humans, DXZ4 in macaque is hypomethylated on the Xi. Notably, a single CpG residue is unmethylated in all male and female pig-tailed macaque clones (Figure 4b, bottom panels). The significance of this is unclear, although it does contribute to the lower overall percentage methylation observed in this species.

### Conserved H3K4me2 banding on the macaque Xi

**Figure 5:**
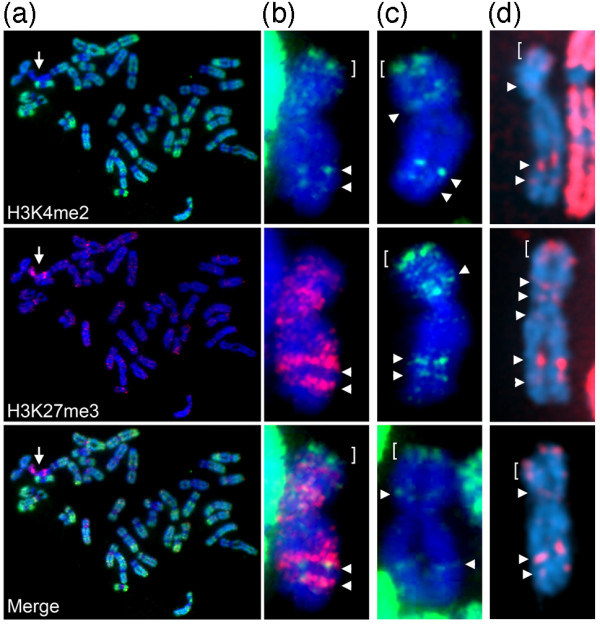
**H3K4me2 distribution on the macaque metaphase Xi**. **(a) **Female rhesus macaque metaphase chromosomes (blue) showing the distribution of H3K4me2 (green) and H3K27me3 (red) by indirect immunofluorescence. The location of the Xi is indicated by the white arrow. **(b) **Female rhesus macaque metaphase Xi (blue) showing the distribution of H3K4me2 (green) and H3K27me3 (red) by indirect immunofluorescence. The tip of the p-arm including the pseudoautosomal region is indicated by the white square bracket. Additional H3K4me2 bands on the Xi are indicated by the white arrowheads. **(c) **Further examples of H3K4me2 band distribution on the Xi to demonstrate reproducibility of the pattern. Signals indicated as for (b). **(d) **Human female telomerase immortalized retinal pigment epithelial cell (hTERT-RPE1) Xi showing the location of H3K4me2 (red) signals, as indicated by the white arrowheads and white bracket.

At metaphase, H3K4me2 is largely absent from the human Xi, with the exception of the tip of Xp including the pseudoautosomal region and DXZ4 midway down Xq [28,29]. Comparing the human H3K4me2 banding pattern to heterochromatin features of the Xi indicates that the euchromatic mark resides at the distal edge of a major band of histone H3 lysine-27 trimethylation (H3K27me3) and macroH2A [28,30,31]. We therefore sought to determine if the banding pattern of H3K4me2 on the macaque Xi resembled that seen in humans. In female rhesus macaque, a single chromosome was consistently marked by bands of H3K27me3 and hypo-H3K4me2 (an example is shown in Figure 5a), features typically characteristic of the Xi [28,30,31]. Close examination of the chromosome revealed an intense H3K4me2 signal midway down the long arm that resided at the distal edge of a major band of H3K27me3 (Figure 5b), and in the vicinity of DXZ4 and *PLS3 *FISH signals (Figure 1a). Additional H3K4me2 signals were observed at the tip of the p-arm, consistent with the pseudoautosomal region, and at the distal edge of a second q-arm band of H3K27me3. H3K4me2 band locations were reproducible on the macaque Xi, consisting predominantly of the major q-arm signal and other weaker bands on the p and q arms (Figure 5c). A similar pattern is observed at the human Xi (compare to Figure 5d), indicating conserved chromatin structure at these regions.

### Euchromatic markers are largely absent from the macaque Xi with the exception of a discrete signal within the interphase territory of the chromosome

**Figure 6:**
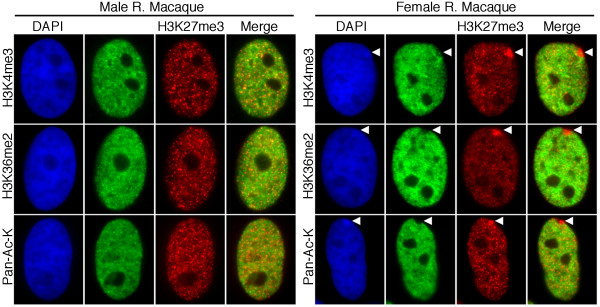
**Distribution of euchromatic chromatin marks relative to the macaque Xi at interphase**. Typical examples of male and female rhesus macaque (R. Macaque) interphase nuclei showing the distribution of H3K27me3 (red), H3K4me3 (green), H3K36me2 (green) and acetylated lysine (Pan-Ac-K, green) as determined by indirect immunofluorescence. Nuclei are counterstained with DAPI (blue). White arrowheads in the female images indicate the location of the Xi.

At interphase, euchromatic markers are absent from the human Xi with the notable exception of a signal within the territory of the Xi [23,28,29] that is inseparable from DXZ4 [29]. Two covalent modifications of histone H3 that are associated with transcription, H3 lysine-4 trimethylation (H3K4me3) and H3 lysine-36 dimethylation (H3K36me2), as well as lysine acetylation (a general euchromatin modification) were examined in rhesus macaque. The three modifications in male cells showed a general nuclear distribution that was absent from the nucleoli and little to no overlap with territories of facultative heterochromatin defined by H3K27me3 (Figure 6, left panels). In female cells, the nuclear distribution was like that of males with the additional observation that the Xi (defined by H3K27me3) lacked signals for the euchromatin markers (Figure 6, right panels). Both H3K4me3 and H3K36me2 showed the presence of a signal within the territory of the Xi, a feature that was occasionally seen for the pan-acetyl lysine antibody.

### Xi DXZ4 in macaque is characterized by H3K4me2 and CTCF

**Figure 7:**
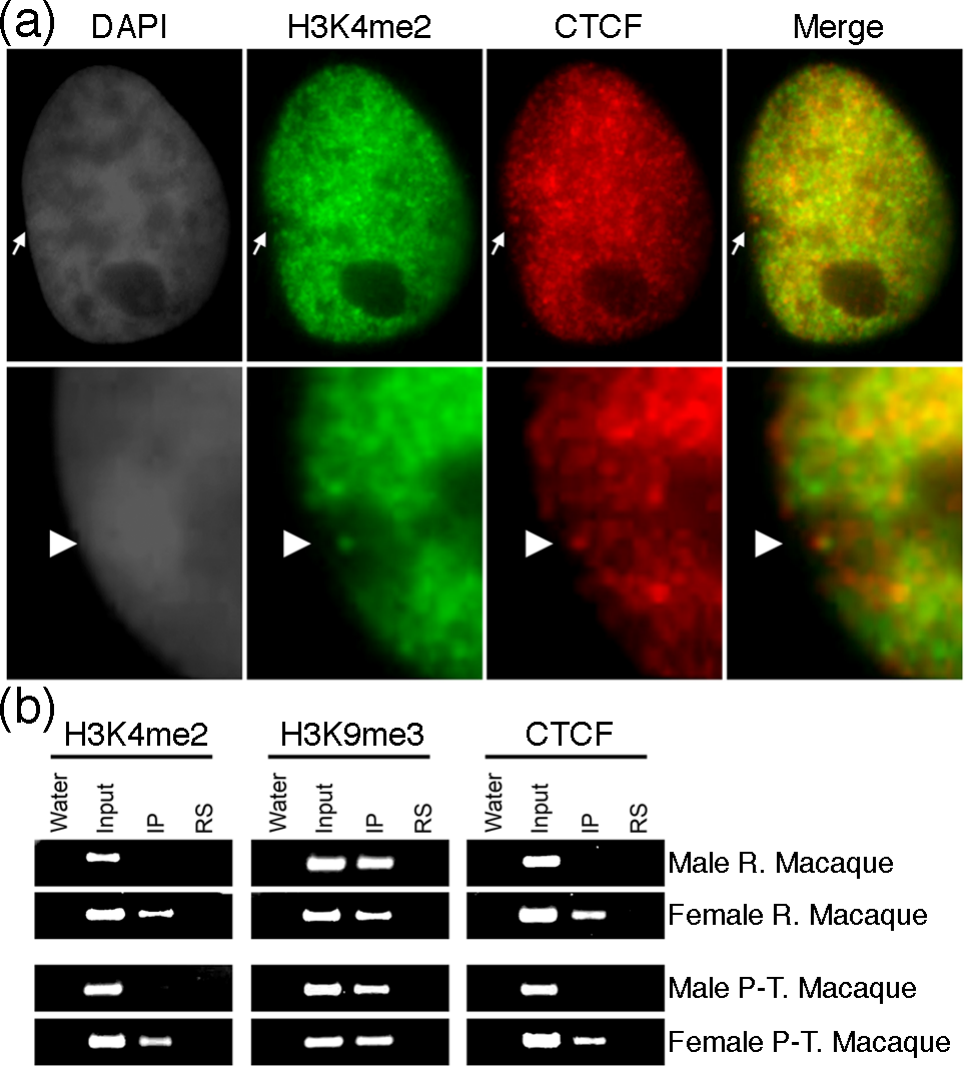
**Female-specific association of CTCF and H3K4me2 with the macaque Xi**. **(a) **Co-localization of CTCF (red) at the H3K4me2 'dot' (green) in the territory of the Xi in a female rhesus macaque nucleus. The white arrow indicates the Xi (top panel). The lower panel shows an independent example zoomed in. The white arrowhead points to the territory of the Xi. Nuclei are counterstained with DAPI, and the collected image converted to gray-scale to emphasize the Barr body. **(b) **Chromatin immunoprecipitation assessing the association of H3K4me2, H3K9me3 and CTCF with DXZ4 in male and female rhesus macaque (R. Macaque) and pig-tailed macaque (P-T. Macaque) chromatin. Samples include a no template control (Water), input DNA (Input), immunoprecipitation with the antibody indicated (IP) and non-specific rabbit serum (RS).

In humans, H3K4me2 is a feature of DXZ4 chromatin on the Xi as is the association of the epigenetic organizer and insulator protein CTCF [12,29]. We sought to determine if CTCF was a feature of macaque DXZ4 Xi. We used H3K4me2 to define the territory of the Xi and location of the DXZ4 dot in female macaque nuclei, and compared this to the nuclear distribution of CTCF. As in humans, CTCF was largely absent from the Xi with the exception of a signal that overlapped with the H3K4me2 'dot' (Figure 7a).

To confirm DXZ4 as the site of H3K4me2 and CTCF, chromatin immunoprecipitation was performed on chromatin prepared from male and female rhesus macaque and pig-tailed macaque, along with the heterochromatin marker H3K9me3. Both male and female samples showed the presence of H3K9me3, whereas H3K4me2 and CTCF were readily detected in the female samples from both species of macaque (Figure 7b). The logical interpretation of these data is that DXZ4 in males and on the Xa is characterized by constitutive heterochromatin, whereas DXZ4 on the Xi is characterized, at least in part, by a euchromatic conformation bound by CTCF, consistent with that seen for human DXZ4 [12].

### Macaque DXZ4 is transcribed

**Figure 8:**
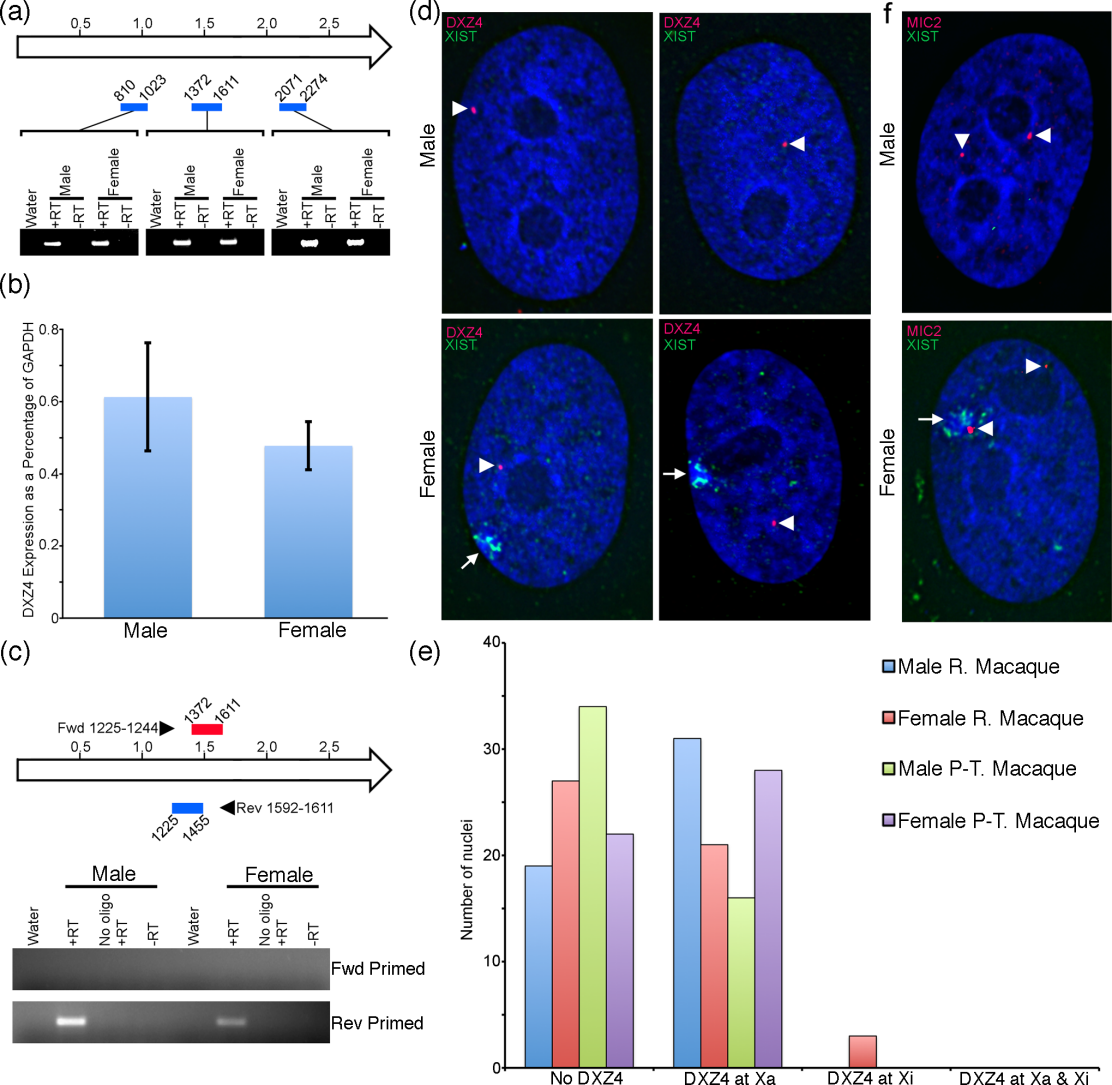
**Expression of DXZ4 in male and female macaques**. **(a) **Schematic representation of a single DXZ4 monomer with positions in kilobases indicated. Below the monomer the blue bars indicate the relative positions of regions of macaque DXZ4 assessed by RT-PCR with the coordinates of the fragment indicated. Each blue bar points to the corresponding RT-PCR data obtained from male and female rhesus macaque cDNA. Samples indicate a no template control (Water) and cDNA prepared form total RNA with (+RT) and without (-RT) including reverse transcriptase enzyme. **(b) **Quantitative RT-PCR (qRT-PCR) analysis of DXZ4 expression in male and female rhesus macaque cDNA. Data represent the mean of four independent triplicate qRT-PCR analyses and the black bar indicates the standard error. Expression levels are represented as a percentage of GAPDH levels. **(c) **Strand-specific RT-PCR of DXZ4. The locations of primers used to prime anti-sense or sense cDNA synthesis are indicated by the right and left-facing arrow-heads and the precise location in DXZ4 is given by the coordinates. The red box represents the region assessed by PCR for anti-sense transcripts and the blue box for sense transcripts. Below the schematic map is the RT-PCR data as an ethidium bromide stained agarose gel. Samples include a no template control (Water), strand-specific primer with reverse transcriptase (+RT), no strand-specific primer with reverse transcriptase (No oligo +RT) and no primer with no reverse transcriptase (-RT). **(d) **RNA FISH analysis of DXZ4 expression in male and female rhesus macaque nuclei. Nuclei are counterstained with DAPI (blue). DXZ4 expression (red) is indicated by the white arrowheads. XIST RNA expression (green) is indicated by the white arrows. **(e) **Quantitative analysis of DXZ4 expression from the Xa and Xi in male and female rhesus macaque (R. Macaque) and pig-tailed macaque (P-T. Macaque) (*n *= 50). **(f) **RNA FISH analysis of *MIC2 *expression (red) relative to XIST (green) in male and female rhesus macaque nuclei.

In humans, DXZ4 is expressed [12]. Transcripts are readily detected from DXZ4 packaged as constitutive heterochromatin in male samples, as well as from the Xi in females, although whether transcripts originate from the heterochromatic or euchromatic portion of the Xi array is unclear. Transcript levels are highly variable between cells. In females DXZ4 can be detected by RNA FISH originating from either the Xa or Xi only, or from both chromosomes simultaneously (Tremblay DC *et al.*, in preparation). We isolated total RNA from male and female rhesus macaque fibroblasts, prepared cDNA and performed PCR with primer sets corresponding to three regions of macaque DXZ4 (Figure 8a). DXZ4 was readily detected in the male and female samples. In order to determine if DXZ4 expression levels varied significantly between males and females, quantitative RT-PCR (qRT-PCR) was performed on the cDNA. Levels of DXZ4 were comparable in the male and female samples analyzed (Figure 8b). In humans, most DXZ4 transcript originates from the sense strand, whereas low levels of anti-sense transcript can be detected originating form the Xi [12]. In order to assess DXZ4 transcript origins, strand-specific cDNA was prepared. In both the male and female samples, transcript could only be detected originating from the sense strand (Figure 8c), contrasting with humans. However, it is important to note that amounts of anti-sense DXZ4 transcript in humans are low, and DXZ4 transcription levels vary significantly between human samples (Tremblay DC *et. al.*, in preparation). Therefore, in order to conclude that macaque does not generate anti-sense transcript, this analysis would need to be extended to numerous other independent macaque samples and cell types. Next we performed RNA FISH with a DXZ4 and XIST (X inactive specific transcript) probe to determine if expression is only from the Xa, or if it can also originate from the Xi. Male macaque samples showed a clear signal for DXZ4 transcripts and no XIST RNA (Figure 8d, top panels). Female macaque samples also showed clear expression of DXZ4 (Figure 8d, bottom panels), although like males almost all transcripts originated from the Xa with only 6% of female rhesus macaque showing expression from the Xi and none in female pig-tailed macaque (Figure 8e). To ensure that this did not reflect an inability to detect DXZ4 transcripts at the Xi, a control experiment was performed using a probe to the pseudoautosomal *MIC2 *gene. *MIC2 *transcripts were detected at the X and Y in males and Xa and Xi in females (Figure 8f). Therefore, while expression of DXZ4 is conserved in macaque, the relevance of the antisense transcript is unclear.

## Discussion

To identify functionally important features of DXZ4, we investigated chromatin structure of the array in the Old World monkey macaque, expression and retention of tandem repeat organization, and primary DNA sequence conservation in a variety of closely and distantly related members of the primate lineage.

Our data indicate that DXZ4 in the great apes and in the Old and New World monkeys is a polymorphic tandem-repeat, with array sizes comparable to those observed in humans [3]. DNA sequence data obtained from a BAC clone also provides evidence of tandem arrangement for the orthologous array in ring-tailed lemurs.

DNA sequence analysis reveals 95 to 97% sequence identity to human DXZ4 in the great apes, 90% in the lesser apes, 87% in Old World monkeys and 77% in New World monkeys. All of these primates had a repeat unit size around 3 kb, high GC content (61 to 65%) and a high incidence of CpG (154 to 192 per monomer). Human DXZ4 contains three internal microsatellite repeats that are the only repeat masked portion of a monomer. Through Old World monkeys the [GGGCC] and [CT] repeats are conserved. The [TAAA] repeat sequence is only conserved in chimpanzee and diverges in the other primates. However, all of the other primates have a simple repeat sequence that is enriched in A nucleotides at this location in the monomer, suggesting that retention of A-rich repetitive DNA is important for this region. In the common marmoset (a New World monkey), all three repeat sequences have diverged, but remain repetitive and retain G-rich, C-rich and A-rich sequence composition, respectively. It is generally accepted that DNA sequence composition influences nucleosome positioning [40]. Close examination of predicted nucleosome occupancy [41] for human DXZ4 using the UCSC genome browser [42] shows that the [TAAA] and [CT] repeats are strongly inhibitory of nucleosome occupancy, whereas the [GGGCC] repeat sequence resides at a peak of nucleosome occupancy (data not shown), suggesting that these sequences influence the position of nucleosomes in the array. Indeed, microarray hybridization using DNA isolated from human chromatin immunoprecipitated with antibodies to H3K4me2 and H3K9me3 revealed well defined peaks of modified nucleosomes predicted to be approximately every fourth nucleosome in DXZ4 [12], suggesting that nucleosome distribution within the array is likely well defined. Therefore, it is tempting to suggest that retention of base composition and location of these repeat sequences in primate DXZ4 is necessary to assist in maintaining nucleosome distribution and chromatin structure within the array.

Conservation of DXZ4 DNA sequence drops off rapidly in the lemurs, galago and tarsier branches of the primate tree. However, sequence homology extends across an approximately 400-bp region of DXZ4 encompassing the bidirectional promoter and binding sites for TFIID and CTCF [12]. Unlike the chromosome 4 macrosatellites RS447 [4] and D4Z4 [6], which both contain ORFs that are conserved through rodents [6], DXZ4 does not obviously encode a protein. Therefore, retention of this sequence is very significant, and suggestive of an important role for DXZ4 in primates as a genomic element, involving CTCF binding and transcription. Preliminary analysis of this region throughout mammals (including mouse) indicates the presence of a tandem repeat downstream of *PLS3 *that shows some homology to this 400-bp sequence, further supporting an important role for this element on the X chromosome (BP Chadwick, unpublished data), and this is now a major focus for our DXZ4 investigation.

Despite a lack of conserved ORFs, DXZ4 is expressed in humans. Most DXZ4 RNA are sense transcripts originating from the Xa, although detectable anti-sense transcription is found specifically in females and therefore likely originates from the Xi [12]. Here we find that DXZ4 is expressed in male and female macaque, although almost all transcription appears to originate form the Xa and no anti-sense transcript was detected. Therefore, expression of DXZ4 is conserved, but the significance of anti-sense transcription remains unclear.

In humans, DXZ4 in males and on the Xa in females is packaged into constitutive heterochromatin characterized by hyper-CpG methylation [3,12], H3K9me3 [12] and HP1γ [32]. Conversely, DXZ4 on the Xi is packaged, at least in part, into euchromatin characterized by H3K4me2, H3K36me2, and histone acetylation, and is hypomethylated at CpG residues and bound by CTCF [3,12,28,29]. Both forms are expressed despite the contrasting chromatin packaging [12]. Here we show that all of these features are conserved at DXZ4 in the Old World monkey macaque. Furthermore, as has been observed in humans [28,30,31], the macaque Xi is characterized by distinct reproducible bands of H3K27me3, with the euchromatic form of DXZ4 located at the distal edge of a major Xq H3K27me3 band. Also consistent with the human Xi [28], additional distinct H3K4me2 signals reside at the distal edge of other H3K27me3 bands, suggesting that conservation of this arrangement has some role in Xi chromatin organization. Therefore, determining the DNA sequence identity of these chromatin elements is a priority.

## Conclusions

These data indicate several conserved features of DXZ4 that are likely important for the organization and function of the array: repeat monomer tandem arrangement; retention of high GC content and CpG incidence; conservation of the internal promoter sequence; conservation of the CTCF binding site; conservation of internal simple repeats; and array expression. Collectively, these features likely contribute to the roles of DXZ4 packaged into constitutive heterochromatin on the Xa and euchromatin bound by CTCF on the Xi. What function DXZ4 has in these contexts remains unclear. However, data from this study highlight important conserved features of DXZ4 that will assist in guiding the formulation of new hypotheses that can be tested to decipher the role of DXZ4 on the X chromosome. Furthermore, elucidation of DXZ4 function on the Xi will likely reveal additional intriguing parallels between the biology of DXZ4 and the contracted form of D4Z4 in FSHD, promoting our appreciation for these enigmatic genomic features.

## Materials and methods

### Cell culture

Human female telomerase immortalized retinal pigment epithelial cells (hTERT-RPE1) were obtained from Clontech (Mountain View, CA, USA). Human male lymphoblastoid cell line GM06992 was obtained from the Coriell Cell Repositories (Coriell Institute for Medical Research), as were the following primate primary fibroblast cells: rhesus macaque (*M. mulatta*) AG08305 (male), and AG08312 (female); pig-tailed macaque (*M. nemistrina*) AG07921 (male), and AG08452 (female); common squirrel monkey (*Saimiri sciureus*) AG05311 (female); black-handed spider monkey (*Ateles geoffroyi*) AG05352 (male). Cells were maintained according to Coriell's recommendations. Female gorilla (*Gorilla gorilla) *lymphoblast cells [43] were a gift from H Willard. Culture media (RPMI for lymphoblasts, and DMEM for fibroblasts), fetal bovine serum and supplements were all obtained from Invitrogen Corp (Carlsbad, CA, USA).

### Metaphase chromosome preparation

In order to enrich for cells in metaphase, growth media of rhesus macaque primary fibroblasts was supplemented with colcemid (Invitrogen) to 25 ng/ml before returning cells to the incubator for an additional hour. Cells were harvested and resuspended in 37°C 75 mM KCl for 15 minutes. To this, one-sixth volume of fixative (three parts methanol to one part acetic acid) was applied before pelleting the cells. Cells were washed an additional six times with fixative, pelleting cells between each wash. Fixed cells were dropped from approximately 30 cm onto cleaned microscope slides resting on damp paper towels on top of a 37°C heat block. Slides were dried at room temperature for an additional 24 hours before use.

### Pulsed field gel electrophoresis, Southern blotting and hybridization

Agarose embedded genomic DNA from primate cells were prepared essentially as described [5].

Agarose embedded DNA was digested with *Xba*I (NEB, Ipswich, MA, USA). Each plug was first equilibrated in 300 μl of 1 × digest buffer at room temperature for 20 minutes, before replacement of buffer with 100 μl of 1 × digest buffer containing 200 units of restriction enzyme. Digests were performed overnight at 37°C. Plugs were loaded onto a 1.0% agarose gel prepared using pulsed field certified agarose (Biorad, Hercules, CA, USA) in 0.5 × TBE. DNA was separated at 13°C in 0.5 × TBE and conditions selected to separate 100 to 400 kb using the auto algorithm function of the CHEF Mapper (Biorad). Markers were loaded in the outer lanes (NEB, MidRange PFG Markers I and II). The gel was then rinsed with water before staining with ethidium bromide (1 μg/ml) at room temperature for 30 minutes. The gel was washed twice with water for 15 minutes each and an image captured. The gel was then treated with 0.25 M HCl for 15 minutes before denaturing for 30 minutes (1.5 M NaCl, 0.5 M NaOH). DNA was transferred to Hybond-N+ (GE Healthcare, Piscataway, NJ, USA) overnight by standard Southern blotting [44]. The membrane was rinsed with 2 × SSC before baking at 120°C for 30 minutes.

A DXZ4 probe was prepared by PCR amplification of regions of human DXZ4 with the following oligonucleotides: CAGGCAGAAATGAGCACCAC and TGGTGGCGGCCATGATCTG (485 bp); ACCAGGCAAACTGCCCAAG and TTCTGGTTTGTCAGGAAGGC (550 bp); ACCCTGTCCTTGGCAGATG and GTTGGACGTAGGCCAGGTG (491 bp); GCCTACGTCACGCAGGAAG and CCAGCGGAAAGTCCATGGG (402 bp); CACTTGGGAGACTCCTGAAC and TGTCCCCGAGGTTGTCTTG (485 bp); TCTCTCGCCCACTTCTACTG and GAGTCGATGGGCCTCTTAG (530 bp). The PCR products were cleaned (Qiagen, Valencia, CA, USA) before labeling with DIG-11-dUTP by random priming (Roche, Basel, Switzerland). The probes were tested for specificity and detection of the anticipated DNA fragment size on a Southern blot of *Eco*RI digested total genomic DNA.

Hybridization was performed overnight at 60°C using Expresshyb (Clontech). Blots were washed the following day at 60°C using two 8-minute washes in 2 × SSC, 0.1% SDS followed by one wash of 8 minutes in 0.2 × SSC, 0.1% SDS. The probe was detected using anti-DIG-alkaline phosphatase, blocking, wash and detection buffers according to the manufacturer's instructions (Roche). Signals were detected by exposure to photographic film (Kodak).

### BAC clone analysis

Human BAC clone 2272M5 was obtained from Invitrogen. Human BAC clone RP11-268A15 was obtained from the Children's Hospital and Research Center at Oakland (CHRCO), as were the following green monkey (*C. aethiops*) and macaque BAC clones: macaque (*M. mulatta*) BAC clones from the CHORI-250 library - CH250-131A6 and CH250-345N15; green monkey BAC clones from the CHORI-252 library - CH252-257K14, CH252-445A22, CH252-338G16 and CH252-199I1. Individual DXZ4 monomers from BAC clones CH250-131A6 and CH252-338G16 were generated by first performing a *Hin*dIII digestion on the BAC clone DNA, gel purifying the 3-kb fragment and cloning into calf intestinal alkaline phosphatase (NEB) treated *Hin*dIII cut pBluescript-II (Agilent Technologies, Santa Clara, CA, USA). Inserts were sequenced on both strands using T7, MM-F1 CCTCTTGATGGCAGTATTGC, MM-F2 CCTGGCCAGCATAGGTCAG, MM-F3 AGAGGCGGCAAGAGAAATGC, SP6, MM-R1 TTGTCAGGAAGGCAGGCTAG, MM-R2 ACATCGGGTTTCCGTCACAG and MM-R3 ATCCAACTTCCACCTCAACG.

### DNA and RNA FISH

For DNA FISH, probes of human BAC clones RP11-268A15 and 2272M5 were labeled with Spectrum Orange or Spectrum Green by nick translation according to the manufacturer's instructions (Abbott Molecular, Abbott Park, IL, USA), followed by ethanol precipitation in the presence of 25 μg of human Cot-1 DNA (Invitrogen) and resuspension in 0.1 ml of Hybrisol VII (MP Biomedicals, Solon, OH, USA). A 1:1 mix of the two probes was denatured at 75°C for 4 minutes, and repetitive sequences blocked at 37°C for 30 minutes before being applied directly to the slide, covered with cover glass, sealed with rubber cement and hybridized for 16 hours at 37°C. Slides were washed at 37°C twice in 50% formamide, 2 × SSC for 8 minutes each, then once in 2 × SSC for 8 minutes before adding ProLong Gold antifade containing DAPI (Invitrogen).

For RNA FISH, a direct-labeled Spectrum Green probe of human XIST exon 1 was prepared as described above and used with a Spectrum Red rhesus macaque 131A6 DXZ4 3-kb subclone probe. Fibroblasts were grown directly on slides before fixing and extracting in 4% formaldehyde, 0.1% Triton-X100 1 × phosphate buffered saline for 10 minutes at room temperature. Slides were washed for 2 minutes each in 1 × phosphate buffered saline before dehydration through 70% and 100% ethanol for 2 minutes each and then air-drying. A 1:1 mix of the BAC and XIST probes was denatured at 72°C for 5 minutes before placing at 37°C for 30 to 60 minutes to block repetitive elements. The probe was applied onto cells and sealed with a coverslip and rubber cement at 37°C for 16 hours in a humidified chamber. Slides were washed twice at room temperature in 50% formamide, 2 × sodium citrate sodium chloride (SSC), followed by 3 minutes at 37°C in 50% formamide 2 × SSC and one wash of 3 minutes at 37°C in 2 × SSC before addition of ProLong Gold antifade containing DAPI (Invitrogen). Control RNA FISH used a Spectrum Red MIC2 BAC clone RP11-1151O1 from Invitrogen.

### Bisulfite sequencing

Macaque genomic DNA was isolated from rheusus macaque and pig-tailed macaque cells using the Blood and Cell Culture DNA Midi-Kit (Qiagen), and bisulfite modified DNA prepared using the EpiTect Bisulfite kit (Qiagen) according to the manufacturer's recommendations. A 621-bp fragment of DNA was PCR amplified from bisulfite-modified DNA using the following primer pair: forward, GGGTATTAGGTAAATTGTTTA; reverse, CCATCCCAAAAACATAATTAAAA. PCR products were TA cloned into pDrive (Qiagen) and individual clones sequenced with M13R.

### Immunofluorescence on interphase cells and metaphase chromosomes

Rabbit polyclonal anti-H3K4me2 (07-030), anti-H3K4me3 (05-745), anti-CTCF (07-729), anti-H3K36me2 (07-274) and anti-acetyl-lysine (06-933) were all obtained from Millipore (Billerica, MA, USA). Mouse monoclonal anti-H3K27me3 (ab6002) was obtained from Abcam (Cambridge, MA, USA). Secondary antibodies were obtained from Jackson ImmunoResearch Laboratories Inc. and Invitrogen (West Grove, PA, USA). Cell staining and preparation of metaphase chromosomes was performed essentially as described [28]. Images were collected using a Zeiss Axiovert 200M fitted with an AxioCam MRm and images managed using AxioVision 4.4 software (Carl Zeiss Microimaging, Inc.).

### Chromatin immunoprecipitation

Chromatin immunoprecipitation using rhesus macaque and pig-tailed macaque cells was performed essentially as described [12]. Antibodies were obtained from Millipore: anti-CTCF (07-729), anti-H3K4me2 (07-030) and anti-H3K9me3 (07-523). Rabbit serum negative control was obtained from EMD (Gibbstown, NJ, USA). Immunoprecipitated DNA was PCR amplified using MM-F1 and MM-R1 (sequences given above).

### RT-PCR, strand-specific RT-PCR and quantitative RT-PCR

Macaque total RNA was isolated from cells using the RNeasy Mini Kit (Qiagen). Residual genomic DNA was removed by pre-treating the RNA with DNaseI (Invitrogen) for 20 minutes at room temperature, before heat inactivating the DNaseI at 70°C in the presence of 2.5 mM EDTA for 15 minutes. cDNA was prepared using 1 μg of total RNA with or without M-MLV reverse transcriptase (Invitrogen) according to the manufacturer's instructions.

cDNA was amplified using Taq polymerase (NEB) with the following cycle: 95°C for 2 minutes, followed by 35 cycles of 95°C for 20 seconds, 58°C for 20 seconds, 72°C for 30 seconds. Amplification used the following primers: MM-F1 and MM-R1; MM-F2 and MM-R2; MM-F3 and MM-R3 (sequences given above).

Strand-specific cDNA was prepared essentially as described above except random hexamers were replaced with a strand-specific primer and an additional control was included of reverse transcriptase without primer. Anti-sense cDNA was primed with MM-F4 (TGACCAAGAGGTCAAAGGCG), whereas sense-strand cDNA was primed with MM-R2. cDNA was assessed with MM-F2 and MM-R2 or MM-F4 and MM-R4 (GTTGGACGTAGGCCAGGTG).

qRT-PCR was performed in triplicate four independent times using random primed cDNA with MM-F2 and MM-R4 using DyNAmo SYBR Green qPCR (NEB) on a CFX96 (Biorad).

## Abbreviations

BAC: bacterial artificial chromosome; bp: base pair; CTCF: CCCTC binding factor; DAPI: 4',6-diamino-2-phenylinole dihydrochloride; FISH: fluorescence *in situ *hybridization; FSHD: facioscapulohumeral muscular dystrophy; H3K4me2: histone H3 dimethylated at lysine 4; H3K4me3: histone H3 trimethylated at lysine 4; H3K9me3: histone H3 trimethylated at lysine 9; H3K27me3: histone H3 trimethylated at lysine 27; H3K36me2: histone H3 dimethylated at lysine 36; HP1γ: heterochromatin protein 1 gamma; kb: kilobase; ORF: open reading frame; qRT-PCR: quantitative reverse transcription polymerase chain reaction; RT-PCR: reverse transcription polymerase chain reaction; SSC: sodium citrate sodium chloride; VNTR: variable number tandem repeat; Xa: active X chromosome; Xi: inactive X chromosome; XIST: X inactive specific transcript.

## Competing interests

The authors declare that they have no competing interests.

## Authors' contributions

BPC conceived of the study, analyzed and interpreted data, performed experiments, and wrote the manuscript. CM carried out experiments and analyzed data.

## References

[B1] Warburton PE Hasson D Guillem F Lescale C Jin X Abrusan G Analysis of the largest tandemly repeated DNA families in the human genome. BMC Genomics 2008 9 533 10.1186/1471-2164-9-533 18992157 PMC2588610

[B2] Bruce HA Sachs N Rudnicki DD Lin SG Willour VL Cowell JK Conroy J McQuaid DE Rossi M Gaile DP Nowak NJ Holmes SE Sklar P Ross CA Delisi LE Margolis RL Long tandem repeats as a form of genomic copy number variation: structure and length polymorphism of a chromosome 5p repeat in control and schizophrenia populations. Psychiatr Genet 2009 19 64 71 10.1097/YPG.0b013e3283207ff6 19672138 PMC4855847

[B3] Giacalone J Friedes J Francke U A novel GC-rich human macrosatellite VNTR in Xq24 is differentially methylated on active and inactive X chromosomes. Nat Genet 1992 1 137 143 10.1038/ng0592-137 1302007

[B4] Kogi M Fukushige S Lefevre C Hadano S Ikeda JE A novel tandem repeat sequence located on human chromosome 4p: isolation and characterization. Genomics 1997 42 278 283 10.1006/geno.1997.4746 9192848

[B5] Tremblay DC Alexander G Jr Moseley S Chadwick BP Expression, tandem repeat copy number variation and stability of four macrosatellite arrays in the human genome. BMC Genomics 2010 11 632 10.1186/1471-2164-11-632 21078170 PMC3018141

[B6] van Deutekom JC Wijmenga C van Tienhoven EA Gruter AM Hewitt JE Padberg GW van Ommen GJ Hofker MH Frants RR FSHD associated DNA rearrangements are due to deletions of integral copies of a 3.2 kb tandemly repeated unit. Hum Mol Genet 1993 2 2037 2042 10.1093/hmg/2.12.2037 8111371

[B7] Bakker E Wijmenga C Vossen RH Padberg GW Hewitt J van der Wielen M Rasmussen K Frants RR The FSHD-linked locus D4F104S1 (p13E-11) on 4q35 has a homologue on 10qter. Muscle Nerve 1995 2 S39 44 10.1002/mus.880181309 7739624

[B8] Deidda G Cacurri S Grisanti P Vigneti E Piazzo N Felicetti L Physical mapping evidence for a duplicated region on chromosome 10qter showing high homology with the facioscapulohumeral muscular dystrophy locus on chromosome 4qter. Eur J Hum Genet 1995 3 155 167 7583041 10.1159/000472291

[B9] Wijmenga C Hewitt JE Sandkuijl LA Clark LN Wright TJ Dauwerse HG Gruter AM Hofker MH Moerer P Williamson R Chromosome 4q DNA rearrangements associated with facioscapulohumeral muscular dystrophy. Nat Genet 1992 2 26 30 10.1038/ng0992-26 1363881

[B10] Tawil R Facioscapulohumeral muscular dystrophy. Neurotherapeutics 2008 5 601 606 10.1016/j.nurt.2008.07.005 19019312 PMC2628543

[B11] Lemmers RJ Wohlgemuth M van der Gaag KJ van der Vliet PJ van Teijlingen CM de Knijff P Padberg GW Frants RR van der Maarel SM Specific sequence variations within the 4q35 region are associated with facioscapulohumeral muscular dystrophy. Am J Hum Genet 2007 81 884 894 10.1086/521986 17924332 PMC2265642

[B12] Chadwick BP DXZ4 chromatin adopts an opposing conformation to that of the surrounding chromosome and acquires a novel inactive X-specific role involving CTCF and antisense transcripts. Genome Res 2008 18 1259 1269 10.1101/gr.075713.107 18456864 PMC2493436

[B13] Saitoh Y Miyamoto N Okada T Gondo Y Showguchi-Miyata J Hadano S Ikeda JE The RS447 human megasatellite tandem repetitive sequence encodes a novel deubiquitinating enzyme with a functional promoter. Genomics 2000 67 291 300 10.1006/geno.2000.6261 10936051

[B14] Snider L Asawachaicharn A Tyler AE Geng LN Petek LM Maves L Miller DG Lemmers RJ Winokur ST Tawil R van der Maarel SM Filippova GN Tapscott SJ RNA transcripts, miRNA-sized fragments, and proteins produced from D4Z4 units: new candidates for the pathophysiology of facioscapulohumeral dystrophy. Hum Mol Genet 2009 18 2414 2430 10.1093/hmg/ddp180 19359275 PMC2694690

[B15] Hewitt JE Lyle R Clark LN Valleley EM Wright TJ Wijmenga C van Deutekom JC Francis F Sharpe PT Hofker M Analysis of the tandem repeat locus D4Z4 associated with facioscapulohumeral muscular dystrophy. Hum Mol Genet 1994 3 1287 1295 10.1093/hmg/3.8.1287 7987304

[B16] Gabriels J Beckers MC Ding H De Vriese A Plaisance S van der Maarel SM Padberg GW Frants RR Hewitt JE Collen D Belayew A Nucleotide sequence of the partially deleted D4Z4 locus in a patient with FSHD identifies a putative gene within each 3.3 kb element. Gene 1999 236 25 32 10.1016/S0378-1119(99)00267-X 10433963

[B17] Dixit M Ansseau E Tassin A Winokur S Shi R Qian H Sauvage S Mattéotti C van Acker AM Leo O Figlewicz D Barro M Laoudj-Chenivesse D Belayew A Coppée F Chen YW DUX4, a candidate gene of facioscapulohumeral muscular dystrophy, encodes a transcriptional activator of PITX1. Proc Natl Acad Sci USA 2007 104 18157 18162 10.1073/pnas.0708659104 17984056 PMC2084313

[B18] Lemmers RJ van der Vliet PJ Klooster R Sacconi S Camaño P Dauwerse JG Snider L Straasheijm KR van Ommen GJ Padberg GW Miller DG Tapscott SJ Tawil R Frants RR van der Maarel SM A unifying genetic model for facioscapulohumeral muscular dystrophy. Science 2010 329 1650 1653 10.1126/science.1189044 20724583 PMC4677822

[B19] Clapp J Mitchell LM Bolland DJ Fantes J Corcoran AE Scotting PJ Armour JA Hewitt JE Evolutionary conservation of a coding function for D4Z4, the tandem DNA repeat mutated in facioscapulohumeral muscular dystrophy. Am J Hum Genet 2007 81 264 279 10.1086/519311 17668377 PMC1950813

[B20] Payer B Lee JT X chromosome dosage compensation: how mammals keep the balance. Annu Rev Genet 2008 42 733 772 10.1146/annurev.genet.42.110807.091711 18729722

[B21] Mohandas T Sparkes RS Shapiro LJ Reactivation of an inactive human X chromosome: evidence for X inactivation by DNA methylation. Science 1981 211 393 396 10.1126/science.6164095 6164095

[B22] Pfeifer GP Tanguay RL Steigerwald SD Riggs AD *In vivo *footprint and methylation analysis by PCR-aided genomic sequencing: comparison of active and inactive X chromosomal DNA at the CpG island and promoter of human PGK-1. Genes Dev 1990 4 1277 1287 10.1101/gad.4.8.1277 2227409

[B23] Boggs BA Cheung P Heard E Spector DL Chinault AC Allis CD Differentially methylated forms of histone H3 show unique association patterns with inactive human X chromosomes. Nat Genet 2002 30 73 76 10.1038/ng787 11740495

[B24] Peters AH Mermoud JE O'Carroll D Pagani M Schweizer D Brockdorff N Jenuwein T Histone H3 lysine 9 methylation is an epigenetic imprint of facultative heterochromatin. Nat Genet 2002 30 77 80 10.1038/ng789 11740497

[B25] Plath K Fang J Mlynarczyk-Evans SK Cao R Worringer KA Wang H de la Cruz CC Otte AP Panning B Zhang Y Role of histone H3 lysine 27 methylation in X inactivation. Science 2003 300 131 135 10.1126/science.1084274 12649488

[B26] Silva J Mak W Zvetkova I Appanah R Nesterova TB Webster Z Peters AH Jenuwein T Otte AP Brockdorff N Establishment of histone h3 methylation on the inactive x chromosome requires transient recruitment of eed-enx1 polycomb group complexes. Dev Cell 2003 4 481 495 10.1016/S1534-5807(03)00068-6 12689588

[B27] Jeppesen P Turner BM The inactive X chromosome in female mammals is distinguished by a lack of histone H4 acetylation, a cytogenetic marker for gene expression. Cell 1993 74 281 289 10.1016/0092-8674(93)90419-Q 8343956

[B28] Chadwick BP Willard HF Cell cycle-dependent localization of macroH2A in chromatin of the inactive X chromosome. J Cell Biol 2002 157 1113 1123 10.1083/jcb.200112074 12082075 PMC2173542

[B29] Chadwick BP Willard HF Chromatin of the Barr body: histone and non-histone proteins associated with or excluded from the inactive X chromosome. Hum Mol Genet 2003 12 2167 2178 10.1093/hmg/ddg229 12915472

[B30] Chadwick BP Variation in Xi chromatin organization and correlation of the H3K27me3 chromatin territories to transcribed sequences by microarray analysis. Chromosoma 2007 116 147 157 10.1007/s00412-006-0085-1 17103221

[B31] Chadwick BP Willard HF Multiple spatially distinct types of facultative heterochromatin on the human inactive X chromosome. Proc Natl Acad Sci USA 2004 101 17450 17455 10.1073/pnas.0408021101 15574503 PMC534659

[B32] Zeng W de Greef JC Chen YY Chien R Kong X Gregson HC Winokur ST Pyle A Robertson KD Schmiesing JA Kimonis VE Balog J Frants RR Ball AR Jr Lock LF Donovan PJ van der Maarel SM Yokomori K Specific loss of histone H3 lysine 9 trimethylation and HP1gamma/cohesin binding at D4Z4 repeats is associated with facioscapulohumeral dystrophy (FSHD). PLoS Genet 2009 5 e1000559 10.1371/journal.pgen.1000559 19593370 PMC2700282

[B33] van Overveld PG Lemmers RJ Sandkuijl LA Enthoven L Winokur ST Bakels F Padberg GW van Ommen GJ Frants RR van der Maarel SM Hypomethylation of D4Z4 in 4q-linked and non-4q-linked facioscapulohumeral muscular dystrophy. Nat Genet 2003 35 315 317 10.1038/ng1262 14634647

[B34] Ottaviani A Rival-Gervier S Boussouar A Foerster AM Rondier D Sacconi S Desnuelle C Gilson E Magdinier F The D4Z4 macrosatellite repeat acts as a CTCF and A-type lamins-dependent insulator in facio-scapulo-humeral dystrophy. PLoS Genet 2009 5 e1000394 10.1371/journal.pgen.1000394 19247430 PMC2639723

[B35] Chadwick BP Macrosatellite epigenetics: the two faces of DXZ4 and D4Z4. Chromosoma 2009 118 675 681 10.1007/s00412-009-0233-5 19690880

[B36] Samonte RV Conte RA Verma RS Localization of human midisatellite and macrosatellite DNA sequences on chromosomes 1 and X in the great apes. J Hum Genet 1999 44 57 59 10.1007/s100380050108 9929980

[B37] ClustalW2 - Multiple Sequence Alignment. http://www.ebi.ac.uk/Tools/msa/clustalw2/

[B38] Rhesus Macaque Genome Sequencing and Analysis Consortium Gibbs RA Rogers J Katze MG Bumgarner R Weinstock GM Mardis ER Remington KA Strausberg RL Venter JC Wilson RK Batzer MA Bustamante CD Eichler EE Hahn MW Hardison RC Makova KD Miller W Milosavljevic A Palermo RE Siepel A Sikela JM Attaway T Bell S Bernard KE Buhay CJ Chandrabose MN Dao M Davis C Delehaunty KD Evolutionary and biomedical insights from the rhesus macaque genome. Science 2007 316 222 234 10.1126/science.1139247 17431167

[B39] Xing J Witherspoon DJ Ray DA Batzer MA Jorde LB Mobile DNA elements in primate and human evolution. Am J Phys Anthropol 2007 Suppl 45 2 19 10.1002/ajpa.20722 18046749

[B40] Segal E Fondufe-Mittendorf Y Chen L Thastrom A Field Y Moore IK Wang JP Widom J A genomic code for nucleosome positioning. Nature 2006 442 772 778 10.1038/nature04979 16862119 PMC2623244

[B41] Dennis JH Fan HY Reynolds SM Yuan G Meldrim JC Richter DJ Peterson DG Rando OJ Noble WS Kingston RE Independent and complementary methods for large-scale structural analysis of mammalian chromatin. Genome Res 2007 17 928 939 10.1101/gr.5636607 17568008 PMC1891351

[B42] UCSC Genome Browser. http://genome.ucsc.edu

[B43] Neubauer RH Rabin H Strnad BC Nonoyama M Nelson-Rees WA Establishment of a lymphoblastoid cell line and isolation of an Epstein-Barr-related virus of gorilla origin. J Virol 1979 31 845 848 92573 10.1128/jvi.31.3.845-848.1979 PMC353513

[B44] Sambrook J Fritsch EF Maniatis T Molecular Cloning: A Laboratory Manual 1989 2 Cold Spring Harbor, NY: Cold Spring Harbor Laboratory Press

[B45] Edgar RC MUSCLE: multiple sequence alignment with high accuracy and high throughput. Nucleic Acids Res 2004 32 1792 1797 10.1093/nar/gkh340 15034147 PMC390337

